# Supplementary figures and images for: The Extract of Arctium lappa L. Fruit (Arctii Fructus) Improves Cancer-Induced Cachexia by Inhibiting Weight Loss of Skeletal Muscle and Adipose Tissue
Source: Nutrients. 2020 Oct 19;12(10):3195. doi: 10.3390/nu12103195 (PMC7603378; doi:10.3390/nu12103195)

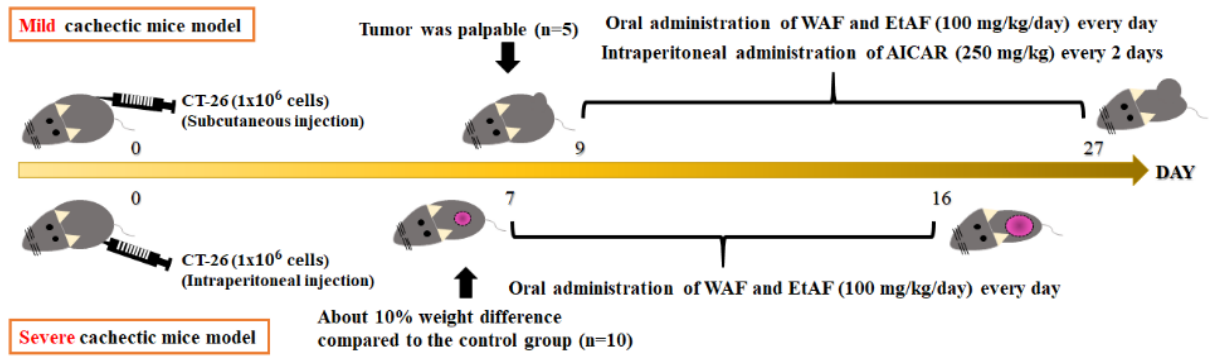

Figure S1. Protocol for mild and severe cachectic mice models by CT-26 cells injection.

Supplement: Supplementary file 1 [file nutrients-12-03195-s001.pdf]
